# Global Community Health Screening and Educational Intervention for Early Detection of Cardiometabolic Renal Disease

**DOI:** 10.5334/aogh.4497

**Published:** 2024-08-21

**Authors:** Natalie L. Nabaty, Tushar Menon, Garrett Trang, Aditya Vijay, Lama Chogyal, Renzo Cataldo, Navin Govind, Pankaj Jain, Priti Singh, Navaz Dolasa, Mandeep Sahani, Prakash Deedwania, Krishnaswami Vijayaraghavan

**Affiliations:** 1 University of Arizona, College of Medicine – Phoenix, AZ, USA; 2 Twinepidemic Inc, Phoenix, AZ, USA; 3 Dalai Lama’s Ladakh Heart Foundation and Research Center, India; 4 Abrazo Arizona Heart Hospital, Pheonix, AZ, USA; 5 Aventyn, Inc.; 6 Banner University Medical Center, Phoenix, AZ, USA; 7 Gila River Health Care, Sacaton, AZ, USA; 8 Desert Kidney Associates, Tempe, AZ, USA; 9 University of California, San Francisco, CA, USA

**Keywords:** cardiometabolic disease, kidney disease, community health intervention, patient education

## Abstract

The global burden of cardiometabolic renal disease is increasing, particularly in underserved communities. Twinepidemic Inc.’s Galvanize Healthy Living program conducts community screenings, risk assessments, and educational interventions globally. We screened 1209 subjects for cardiovascular-kidney-metabolic syndrome, assessing their disease knowledge and self-confidence. Mean age was 50, with 65% females and 35% males. Imaging post-risk assessment revealed abnormalities: EKG (16%), echocardiogram (10%), carotid plaque (9%), ABI (2.5%), and eye exam (3.6%, including 8 retinopathies, 14 cataracts). New onset DM was found in 8%, prediabetes in 18.5%, High LDL in 4.2%, low HDL in 40.2%, high triglycerides in 13.1%, and abnormal BP in 38%. In addition, 18.2% were reclassified to a higher category of risk levels after imaging. Significant improvements in knowledge and self-empowerment (all *p* < 0.001) were seen after educational interventions. This study underscores early risk assessment’s potential to enhance health outcomes globally for underserved populations, validating POC imaging and emphasizing the role of accessible care and education in patient engagement and empowerment.

## Background and Objectives

Cardiovascular disease (CVD) is the leading cause of death, affecting more than 20.5 million people annually around the world [1], and 85% of those deaths are in low- and middle-income countries (LMICs) [2]. CVD, metabolic syndrome, and renal disease are increasingly being considered in a trifecta, due to their shared risk factors and linked pathophysiology. Risk factors of cardiovascular-kidney-metabolic (CKM) syndrome are just as prevalent, if not more so, than in high-income countries [1, 3, 4]. The heart and kidneys share similar vascular risk factors such as diabetes mellitus (DM), hyperlipidemia (HLD), hypertension (HTN), metabolic syndrome, and smoking. They are linked in pathology through cardiorenal syndromes, thus emphasizing the importance of primary—and even primordial—prevention for these systemic illnesses [5, 6].

Cardiovascular-kidney-metabolic (CKM) syndrome is a systemic disorder involving interactions among metabolic risk factors, chronic kidney disease (CKD), and the cardiovascular system, leading to multiorgan dysfunction and adverse cardiovascular outcomes. Its increasing prevalence poses a significant public health challenge. In the United States, about 40% of adults are obese, and nearly half have some form of cardiovascular disease (CVD), including hypertension [7]. Metabolic syndrome, affecting about one-third of adults, significantly increases the risk of CVD, and CKD and is associated with higher rates of heart failure, coronary artery disease, and mortality [8]. Diabetes, impacting more than 10% of adults, is a leading cause of CKD, with about 40% of diabetics developing CKD [7]. CKM syndrome, encompassing obesity, diabetes, CKD, and CVD, leads to significant healthcare costs and reduced quality of life. CKD exacerbates CKM syndrome by increasing the risk of hypertension, anemia, and systemic inflammation, all of which deteriorate cardiac function [8, 9]. Vascular calcification, a complication of CKD, correlates with ischemic events, including myocardial infarction and peripheral artery disease [8].

Suboptimal CKM health results in premature mortality, multiorgan dysfunction, and substantial healthcare costs, primarily due to CVD [9]. CKM syndrome increases the risk of coronary heart disease, stroke, heart failure, peripheral artery disease, atrial fibrillation, and sudden cardiac death [9]. Addressing the high prevalence of these risk factors necessitates early detection, lifestyle modification, and targeted pharmacotherapy, to manage and prevent the progression of CKM-related conditions [9]. Additional risk factors for CKM syndrome include adverse social determinants of health, mental health disorders, sleep disorders, and sex-specific risk enhancers such as premature menopause and adverse pregnancy outcomes [9]. The prevalence of poor CKM health is particularly high among historically marginalized populations, who have limited access to healthy lifestyles and self-care, often driven by socioeconomic, political, and environmental factors [8, 9]. Enhanced screening, prevention, and management approaches are critical to mitigating the impact of CKM syndrome on public health [9].

There has been a recent paradigm shift in preventing cardiometabolic disease with primary prevention, which targets risk factors after they develop, to primordial prevention, which focuses on preventing the development of risk factors for diseases before they occur [10]. Despite its importance, primordial and primary prevention screening is only recently gaining traction in LMICs.

Previous community health screenings in LMICs have demonstrated significant successes and challenges in detecting and managing various CKM syndrome–related diseases. For instance, screenings in urban Karachi identified a high prevalence of metabolic syndrome (MetS) among high-risk individuals, with 54.9% meeting the International Diabetes Federation criteria and 55.4% meeting the US National Cholesterol Education Program criteria [11]. Factors like obesity (OR = 2.14), age >44 years (OR = 2.64), and family history of diabetes (OR = 1.71) were significant predictors [11]. Similarly, screenings for chronic kidney disease (CKD) in low-/middle-income countries have highlighted the potential of early intervention, despite challenges in implementation and cost-effectiveness [12]. Cardiovascular disease (CVD) screenings and community-based interventions in these regions have shown success in increasing population knowledge and influencing lifestyle changes, although consistency in reducing smoking and alcohol consumption remains an issue [13, 14]. The overarching challenge across these screenings is the limitation posed by resource constraints, low participation rates, and lack of follow-up care, which can undermine the long-term effectiveness of these programs [11–14].

Point-of-care testing (POCT) has shown transformative potential in the management of CKM syndrome, particularly in detecting chronic kidney disease (CKD) early and customizing treatments for diabetes and atherosclerotic cardiovascular diseases. POCT is especially valuable in remote or low-resource settings where healthcare access is limited [15], and its affordability and accessibility help address healthcare disparities among underserved communities.

Twinepidemic Inc., a nonprofit organization, through its Galvanize Healthy Living (GHL) program and collaboration with other nonprofits, performs community screening and early risk assessment locally and internationally. The GHL program aims to improve health outcomes for those at risk of cardiometabolic renal disease by increasing access to affordable care, facilitating the availability of newer technology, and embracing local belief systems to serve the underserved and reduce the global burdens of disease and healthcare costs.

## Methods

The community events were broadcast to their respective communities through social media, word of mouth, direct patient marketing, organizational websites, and radio and newspaper advertisements. Patients signed informed consent forms and completed registration and demographic data collection. Before any medical tests, patients underwent multiple pre-screening surveys, including a Risk Questionnaire (RQ), Knowledge Assessment Test (KAT), and a Self-Assessment Questionnaire (SAQ) to establish the patients’ baseline knowledge of the various aspects of heart disease, kidney disease, and DM, as well as their confidence in implementing necessary lifestyle modifications [16]. Next, a biometric assessment consisting of BP, weight, waist circumference, impedance-derived body fat, muscle mass, body water and bone density, BMI, and a blood draw consisting of point-of-care testing for A1c, blood glucose, lipids, and serum creatinine were performed. A pre-screening, one-lead EKG was performed to detect any cardiac abnormalities. Patients with a positive screening were sent to have a 12-lead EKG.

Based on the results from the biometric data, blood tests, and initial baseline EKG, a risk assessment score was determined, and individuals were classified into various risk categories, including low, low to intermediate, intermediate, intermediate to high, high risk, and extremely high risk. In individuals who were classified as above low risk, further testing was performed, including 12-lead EKG, ECHO, ABI (ankle-brachial index), carotid artery ultrasound, COVA (a single-lead EKG and intracardiac pressure monitoring necklace), retinal disease screening, neuropathy screening, and Fibroscan score. After this additional testing, a reclassification of risk was performed, with the possibility that the patients’ risk category might be adjusted based on the results.

On site, patients participated in a consultation with a physician who provided education on risk assessment and management strategies. Physicians ranged in specialty from family medicine to endocrinology, cardiology, and nephrology. Volunteers helped participants enroll in educational workshops on chronic disease management. To further encourage patients, they were offered coupons for coronary artery calcium scores, discounts on healthy foods from local supermarkets, and coupons for gym memberships.

Finally, a post-screening assessment was performed 4 weeks later. This included retaking the KAT and SAQ and comparing these results to their baseline results. Ninety-day and yearly telephone/text follow-ups for outcomes were performed to track adherence and outcomes of cardiovascular/metabolic/renal events that required hospitalization. A flow chart diagram of the steps of the methods is available in Appendix A.

### Statistical methods

SPSS and MEDCALC were used for data analysis. Patient demographics and other baseline characteristics were summarized using descriptive statistics. Screening laboratory values, as reported, were summarized with standard descriptive statistics. The numbers and percentages of all eligibility criteria at screening were provided. The number and percentage of patients with pre-existing risk variables, prevalence of imaging findings, and detection rate of risk factors were presented using standard statistical assessment and presentation modules.

Continuous variables were summarized using *n*, mean, median, and standard deviation, and minimum, maximum, and categorical variables were summarized using frequency and percentage. Different exposure groups and pre- and post-test scores were compared using the chi-square test for categorical variables or the *t*-test for continuous variables. The *p*-values were provided for statistical significance.

### Ethical approval

Our original research paper was conducted in accordance with ethical principles, ensuring the welfare, rights, and confidentiality of all participants involved. The study underwent rigorous review and received approval from the IRB representative, Valerie Golembiewski, prior to commencement, affirming its adherence to established ethical standards. Her details are below:

Valerie Golembiewski, Argus IRB Inc.

6668 S Hidden Flower Way

Tucson, AZ 85756

+1 (520) 298-7494

argusirb@juno.com

## Results

A total of 1,209 subjects have been screened so far. Data were entered using Excel and then transferred into the statistical programs utilized by the data analyst (SPSS, MEDCALC). At baseline, the mean age was 50; 65% were females; the mean BP was 126/82; the mean body fat was 24%; A1c was 5.4 mg/dL; LDL was 73; HDL was 42; and triglycerides were 85. Six percent were current smokers, and 9% were past smokers. Less than 1% had pre-existing heart disease. Diabetes was noted in 8%, hypertension in 24%, and CKD in 4.5% (see Table 1). Family history was positive for heart disease in 9.2%, diabetes in 10.7%, and hypertension in 36% (see Table 1).

**Table 1:** Demographic Characteristics and Significant Medical and Family Histories.

CHARACTERISTIC	*N*(%)	MIN–MAX	MEAN ± SD	SEM
Age	1209(100)	10–94	51 ± 16	0.4
SBP	1201(99)	11–220	126 ± 21	0.6
DBP	1197(99)	47–126	82 ± 12	0.3
Body fat	1076(89)	11–50	24 ± 4	0.1
Bone density	1078(89)	0.6–33	3 ± 2	0.06
Body water	1079(89)	0.5–80	53 ± 8	0.2
BMI	1092(90)	14–51	25 ± 4	0.1
A1c	567(47)	4.1–10.3	5.5 ± 0.7	0.03
Blood Glucose Fasting	419(35)	57–298	96 ± 24	1
Total cholesterol	614(51)	99–275	142 ± 32	1
LDL	601(50)	21–216	77 ± 26	1
HDL	614(51)	3–95	44 ± 14	0.5
Triglycerides	611(50)	30–483	100 ± 58	2
Serum creatinine	536(44)	0.5–5	0.9 ± 0.3	0.01
eGFR	536(44)	10–130	90 ± 18	0.8
Fibroscan score	17(1.4)	4–18	6 ± 3.4	0.8
ECHO:LVEF	145(12)	24–79	62 ± 8	0.7
IVSD	143(12)	0.6–2	1 ± 0.2	0.02
LA diameter	137(11)	0.8–6	4 ± 0.7	0.06
LVPWD	143(12)	0.6–4	1 ± 0.6	0.05
MV dec time	143(12)	68–583	233 ± 65	5
South Asian/Indian from Ladakh*	595(49)	–	–	–
South Asian/Indian from Phoenix	206(17)	–	–	–
Hispanic Americans	97(8)	–	–	–
Black American	97(8)	–	–	–
Caucasian American	20(2)	–	–	–
Smoker current	31(6)	–	–	–
Smoker past	48(9)	–	–	–
Hx heart disease	5(1)	–	–	–
Hx stents	2(<1)	–	–	–
Hx bypass surgery	3(<1)	–	–	–
Hx stroke	1(<1)	–	–	–
Hx PVD	4(<1)	–	–	–
Hx A–fib	2(<1)	–	–	–
Hx DM	64(8.2)	–	–	–
Hx HTN	204(24)	–	–	–
Hx CHF	1(<1)	–	–	–
Hx CKD	12(4.5)	–	–	–
Family History of MI	78(9.2)	–	–	–
Family History of DM	90(11)	–	–	–
Family History of HTN	306(32)	–	–	–
Family History of Stroke	none	–	–	–

Baseline Characteristics of screened subjects and their pre-existing risk factors.

* Characterizing the ethnic boundaries of our heterogeneous population was challenging. In India, ethnic identity is often shaped by religious affiliation rather than by racial boundaries, with individuals identifying as Hindu, Muslim, Sikh, Christian, Jain, or Buddhist. Additionally, we served groups such as Tibetan refugees and Nepali Hindus in Ladakh. This religious identification spans diverse linguistic, regional, and cultural practices, illustrating India’s vast diversity but defying strict categorization of nationality or ethnicity.

Abbreviations: ECHO:LVEF, echocardiogram-derived left ventricular ejection fraction; IVSD: interventricular septal thickness end-diastole; LVPWD: left ventricular posterior wall dimensions; MV dec time: mitral valve deceleration time.

**Figure 1:**
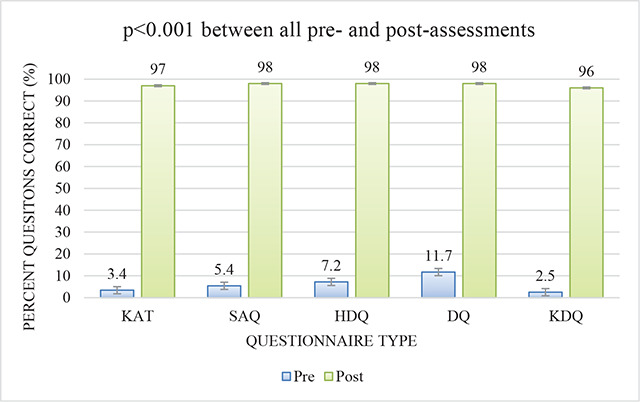
Patient Pre- and Post-test Scores after Educational Intervention. Graphical depiction comparing patient empowerment pre- and post-educational intervention, highlighting statistically significant improvement after intervention. Abbreviations: KAT: Knowledge Assessment Test; SAQ: Self-Assessment Questionnaire; HDQ: Heart Disease Questionnaire; DQ: Diabetes Questionnaire; KDQ: Kidney Disease Questionnaire

After the initial risk assessment, imaging was performed. This revealed abnormal EKG in 16%, abnormal ECHO in 10%, abnormal carotid plaque in 9%, abnormal ABI in 2.5%, and abnormal eye exams in 3.6% (8 retinopathies, 14 cataracts) (see Table 2). New-onset diabetes was detected in 8%, prediabetes in 18.5%, elevated LDL in 4.2%, low HDL in 40.2%, elevated triglycerides in 13.1%, and abnormal systolic or diastolic BP in 38% (see Table 3). Following imaging, a reclassification to a higher level of risk compared to the patients’ baseline risk was demonstrated in 18.2% of subjects (see Table 4). Figure 1 shows that post-intervention responses were highly significant, demonstrating a positive effect on patient knowledge and self-empowerment (*p* < 0.001). In our observed cohort, two subjects needed pacemaker placement: one patient from Ladakh and another South Asian patient from Phoenix. Two subjects needed valvular surgery due to their severe valvular heart disease (VHD); both patients were from Ladakh and sent to Delhi for further medical care. Three patients needed stent procedures: two were South Asians from Phoenix, and one was from Ladakh. Two subjects needed coronary artery bypass grafting (CABG): one patient from Pheonix and one from Ladakh. One patient from Ladakh needed atrial septal defect (ASD) repair for a large ASD. Forty-four patients had abnormal echocardiogram findings. Lastly, two patients were diagnosed with hypertrophic cardiomyopathy (HOCM); both were Black Americans from Pheonix.

**Table 2:** Prevalence of Imaging Findings.

IMAGING	CASES/*N* (%)
EKG	72/452 (16)
TTE	20/222 (10)
Carotid US> 20% plaque	7/77 (9)
ABI	2/77 (2.5)
Eye exam	22/596 (3.6)
Fibroscan	0/16 (0)
Impedance cardiography	7/98 (8)

Prevalence of abnormal findings on imaging of the screened patients after initial risk stratification. Of the 22 subjects that had an abnormal eye exam, 8 patients had retinopathy, and 14 had cataracts.

**Table 3:** Detection Rate of DM, HLD, and HTN.

RISK FACTOR	CASES/*N* (%)
Abnormal A1C	150/567 (26.5)
Prediabetes	105/567 (18.5)
New onset Diabetes	45/567 (8)
Abnormal LDL	25/601 (4.2)
Abnormal HDL	250/614 (40.7)
Abnormal Triglycerides	80/611 (13.1)
Abnormal SBP	266/1201 (22)
Abnormal DBP	657/1197 (54.9)

Detection rate of DM, HLD, and HTN (known risk factors for CKM syndrome) during the screening event.

**Table 4:** Reassessment of CKM Syndrome Risk Category.

	BASELINE RISK	PERCENT RECLASSIFIED AFTER IMAGING					
**Risk category**	**–**	**Very Low**	**Low**	**Intermediate**	**High**	**Very High**	**Extreme**
Very Low	1%	0	0.5	0.5	0	0	0
Low	80%	–	71.4	8	1	0	0
Intermediate	15%	–	–	6.6	6	2	0
High	3%	–	–	–	2		0.82
Very High	1%	–	–	–	–	1	0
Extreme	0	–	–	–	–	–	0

Reclassification of cardiometabolic risk after imaging during the screening event. (*N* = 1209)

## Discussion

This community-based cohort screening was conducted at the Dalai Lama’s Ladakh Heart Foundation and Research Center in Leh, India; African American and Hispanic communities in South Phoenix; and South Asian communities in the Indo-American Cultural center of Phoenix. The GHL program demonstrated that screening for CKM syndrome is feasible and necessary, particularly for under-resourced settings. Using POCT enabled effective screening in areas without traditional infrastructure for lab work. Key findings included the detection of new-onset pre-diabetes in 18.5%, new-onset diabetes in 8%, elevated LDL in 4.2%, low HDL in 40.2%, high triglycerides in 13.1%, and new-onset systolic and diastolic hypertension in 38% of the patients screened. The rising incidence of cardiometabolic disease at younger ages highlights the need for primordial and primary prevention across the lifespan [17, 18]. Individuals with CKD have a greater chance of acquiring CVD than they have of acquiring end-stage renal disease [19]. The ADVANCE trial (2008) of ~11,000 patients showed the close relationship between CKD, CVD, and DM, demonstrating that thorough glucose control leading to a hemoglobin A1c of 6.5% significantly reduced microvascular events, by about 20% [20]. One study found a twofold increase in the risk of atrial fibrillation, stroke, and coronary and peripheral artery diseases in individuals with eGFR (<60 mL/min/1.73 m²) [21]. Additionally, screening in underserved populations is imperative, shown by the increasing global burden of CVD, CKD, and DM [22, 23]. Ultimately, the goal of primordial prevention is to promote healthy behaviors and optimal cardiovascular health from childhood onward to reduce the burden of CKM syndrome.

In our screening study, we used advanced POC imaging modalities, including POC ECHO, COVA, and FibroScan, for comprehensive health assessments. Our findings validated POC imaging for reclassifying cardiometabolic renal disease risk, identifying higher risk in 18% of subjects with ultrasound imaging after initial testing. Abnormal EKGs were noted in 16%, abnormal ECHO in 10%, carotid plaque in 9%, abnormal ABI in 2.5%, and abnormal eye exam in 3.6%. Managing risk factors such as weight, obesity, insulin resistance, dyslipidemia, sodium intake, physical activity, and smoking is crucial to mitigating cardiometabolic renal disease. Despite this, medical management of these conditions with beta-blockers, antiplatelets, and statins remains suboptimal worldwide, and this disparity is exacerbated in those with underlying CKD, further highlighting the need to focus on lifestyle modification and primordial prevention [24]. The detection of risk factors enabled an aggressive strategy for lifestyle modification as well as pharmacological, procedural, and surgical interventions. Ultimately, it allowed us to deliver tailored healthcare recommendations to our study participants.

Global health disparities also exist in the availability and affordability of key therapeutics for CKM syndrome. For example, a comparative study showed that roughly 3 out of 10 patients diagnosed with hypertension met blood pressure goals within LMICs, with factors such as the lack of adherence, availability, and affordability cited as barriers [25]. Another study found that only 1 in 10 people in LMICs who were eligible for statins were actually using them for primary prevention [26]. For patients from Phoenix who scored at intermediate risk or above, coupons for CAC assessment at a local imaging site were provided after they had completed the program, facilitating early intervention and cardiovascular event reduction. The MESA study validated CAC data, showing higher total event rates (8% and 12%) for participants with CAC scores of 101–300 and >300, respectively; for CVD 4% and 8%, respectively; for CHD 8% and 15%, respectively; for death after 8-year follow-up; and close to a 10-year risk of ≥20% [27]. All of this underscore the need for risk factor modification to the same extent in secondary-prevention patients.

Few studies have comprehensively screened for primordial risk factors of cardiometabolic renal disease in global settings while also including combining patient education and empowerment. A meta-analysis by Heine et al. significant impacts disease knowledge, attitude, and behavior in LMICs through health interventions for DM [28]. The PURE study highlighted worse cardiovascular outcomes in patients from LMICs due to poorer healthcare despite their better risk factor profiles and also highlighted that this disparity was exacerbated by lower education levels [29]. Our program included an educational component to try to mitigate this disparity.

The Framingham risk score, pooled risk equation, Q Risk, and other risk-scoring tools mainly focus on CV events, whereas the Galvanize Healthy Living Score targets comprehensive cardiometabolic renal risk assessment with early detection and patient engagement. Responses between the pre- and the post-assessments showed significant improvement (KAT, *p* < 0.0001; SAQ, *p* < 0.001; HDQ, *p* < 0.001; DQ, *p* < 0.001; KDQ, *p* < 0.001) in patient knowledge and empowerment. The event modeled a one-stop-shop approach for result dissemination and patient engagement facilitated by an interdisciplinary team of specialists and generalists, from family medicine to nephrology, endocrinology, and cardiology. There are still gaps in non-physician-based interventions to increase adherence to treatment and lifestyle changes for CKM syndrome–related risk factors [13, 14]. Additionally, programs must continually practice cultural humility to cultivate acceptability of belief systems and to increase patient empowerment over their own health.

Limitations of our study include a heterogeneous population and a lack of event calculation since the design was not a long-term prospective cohort study of the same populations. The study was intended to be a point-of-care cross-sectional screening program, and the reported results are an effort to engage patients early, rather than having delay intervention until an event occurs. Additionally, not all data points were available due to logistical challenges in providing screening/testing in different global locations. These affect the generalizability of the findings to other ethnic groups.

## Conclusions

The Galvanize Healthy Living programs innovate healthcare by blending clinical expertise with a thorough understanding of human experience. Recognizing conditions like diabetes and heart disease as real-life challenges, these programs prioritize education to empower individuals in managing their health, and their commitment extends beyond traditional care, advocating for accessible, quality healthcare as a universal right. By leveraging advanced technology, they enhance patient care with timely, effective interventions. Their holistic approach and outreach to underserved communities highlight their mission to create an inclusive, compassionate healthcare system tailored to diverse needs.

Learning Objectives

1. Define *primordial, primary, secondary*, and *tertiary* prevention, and discuss the impact of early detection through community health screening.
2. Recognize the importance of patient engagement and empowerment through early risk assessment strategies.
3. Explain the magnitude of comorbidity at the cardiometabolic-renal intersection and opportunities to prevent the progression of disease.
4. Translate the results of a screening program to reduce inequalities.

## Clinical Perspectives

This study emphasizes the need to expand primordial screening for CKM syndrome globally, especially in LMICs. Patients in LMICs benefit from POC testing, education, and affordable healthcare access, enabling risk factor modification. While current practice focuses on primary prevention, our study suggests a greater emphasis on primordial prevention—preventing the development of risk factors. Ideally, this would involve widespread screening programs in rural and underserved areas, triaging individuals for immediate healthcare, and educating others on health maintenance. Implementing community-based screening and education initiatives can facilitate early intervention and reduce disease burden. The study highlights the need for proactive healthcare strategies to combat the growing epidemic of cardiometabolic renal disease.

## Data Availability

The data underlying this study are included in the published article and its supplementary files. Further details and additional data are available from the corresponding author, Krishnaswami Vijayaraghavan (kvijaymd@gmail.com), upon request. Please note that certain data may be restricted due to privacy and ethical considerations.

## Additional File

The additional file for this article can be found as follows:
