# Supplementary figures and images for: Global Community Health Screening and Educational Intervention for Early Detection of Cardiometabolic Renal Disease
Source: Ann Glob Health. 2024 Aug 21;90(1):54. doi: 10.5334/aogh.4497 (PMC11342830; doi:10.5334/aogh.4497)

## Appendix A

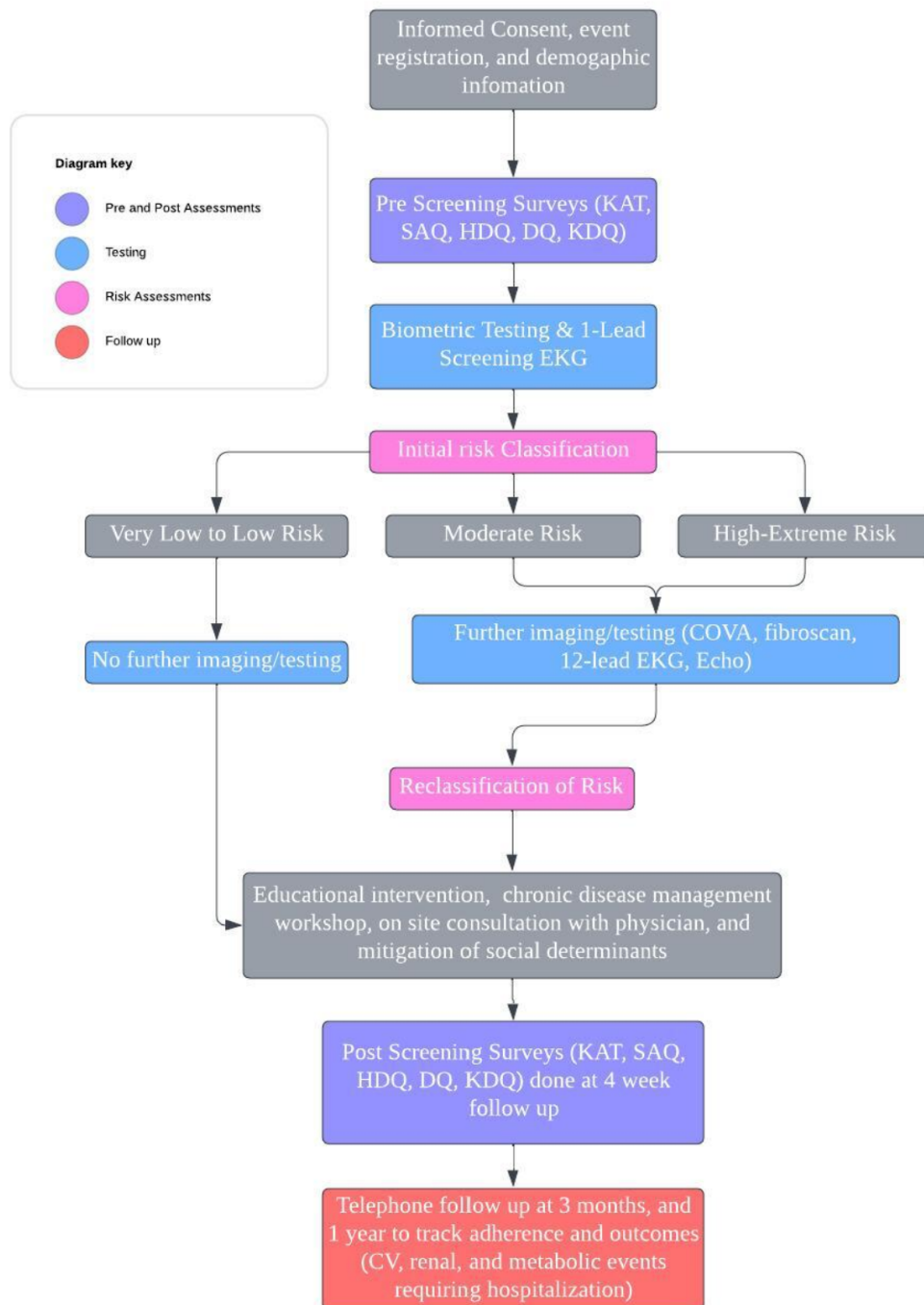

Supplement: Supplementary material. — Appendix A. [file agh-90-1-4497-s1.pdf]
